# SGLT2 inhibition, plasma proteins, and heart failure: a proteome-wide Mendelian Randomization and colocalization study

**DOI:** 10.3389/fcvm.2024.1371513

**Published:** 2024-04-25

**Authors:** Jinlan Luo, Lili Shi, Jingrui Liu, Gen Li, Ling Tu, Shuiqing Hu

**Affiliations:** ^1^ Department of Geriatric Medicine, Tongji Hospital, Tongji Medical College, Huazhong University of Science and Technology, Wuhan, China; ^2^ Hubei Key Laboratory of Genetics and Molecular Mechanisms of Cardiological Disorders, Wuhan, China; ^3^ Division of Cardiology and Department of Internal Medicine, Tongji Hospital, Tongji Medical College, Huazhong University of Science and Technology, Wuhan, China; ^4^ Department of Cardiothoracic and Vascular Surgery, Tongji Hospital, Tongji Medical College, Huazhong University of Science and Technology, Wuhan, China

**Keywords:** sodium-glucose cotransporter 2, heart failure, circulating protein, Mendelian Randomization, LRRTM2

## Abstract

**Objective:**

To investigate the causal contributions of Sodium-glucose cotransporter 2 (SGLT2) inhibition on Heart Failure (HF) and identify the circulating proteins that mediate SGLT2 inhibition's effects on HF.

**Methods:**

Applying a two-sample, two-step Mendelian Randomization (MR) analysis, we aimed to estimate: (1) the causal impact of SGLT2 inhibition on HF; (2) the causal correlation of SGLT2 inhibition on 4,907 circulating proteins; (3) the causal association of SGLT2 inhibition-driven plasma proteins on HF. Genetic variants linked to SGLT2 inhibition derived from the previous studies. The 4,907 circulating proteins were derived from the deCODE study. Genetic links to HF were obtained through the Heart Failure Molecular Epidemiology for Therapeutic Targets (HERMES) consortium.

**Results:**

SGLT2 inhibition demonstrated a lower risk of HF (odds ratio [OR] = 0.44, 95% CI [0.26, 0.76], *P* = 0.003). Among 4,907 circulating proteins, we identified leucine rich repeat transmembrane protein 2 (LRRTM2), which was related to both SGLT2 inhibition and HF. Mediation analysis revealed that the impact of SGLT2 inhibition on HF operates indirectly through LRRTM2 [β = −0.20, 95% CI (−0.39, −0.06), *P* = 0.02] with a mediation proportion of 24.6%. Colocalization analysis provided support for the connections between LRRTM2 and HF.

**Conclusion:**

The study indicated a causative link between SGLT2 inhibition and HF, with plasma LRRTM2 potentially serving as a mediator.

## 1 Introduction

Sodium-glucose cotransporter 2 (SGLT2) is predominantly located in the epithelial cells of the kidney's proximal tubule (1). This protein accounts for daily glucose reabsorption in the body. SGLT2 inhibitors, encompassing dapagliflozin, empagliflozin, and canagliflozin are innovative oral hypoglycemic agents. However, they are appreciated not just for their ability to target SGLT2, thereby impeding glucose reabsorption, but also for their nephroprotective and cardioprotective benefits, including heart failure (HF) (2, 3). Heart failure, a multifaceted clinical syndrome arising from cardiac overload and injury and marked by common signs including exhaustion, edema in the ankles, and dyspnea, now affects over 64 million people globally, resulting in substantial morbidity and mortality (4–6).

Multiple large-scale randomized clinical trials have supplied compelling evidence supporting the positive effects of SGLT2 inhibitors on HF, irrespective of diabetes status. The EMPA-REG OUTCOME trial involving empagliflozin was the pioneering study to demonstrate significant cardioprotective advantages among patients with type 2 diabetes. A striking 35% reduction in HF hospitalizations ignited scientific interest in the potential of SGLT2 inhibition for treating HF (7). The CANVAS Program, which employed canagliflozin, further substantiated this concept, indicating a substantial 33% decrease in hospitalizations for HF among those receiving canagliflozin (8). Compared to a placebo, canagliflozin exhibited a decreased incidence of death or hospitalization [HR, 0.70; 95% CI (0.55–0.89)] (9). Similarly, in the DECLARE-TIMI 58 trial that occupied Dapagliflozin, analogous reductions in HF hospitalizations were observed in both HfrEF and HFpEF patients (10). Moreover, the SGLT2 inhibitor canagliflozin was proven to postpone the elevation in N-terminal pro–B-type natriuretic peptide (NT-proBNP) and high-sensitivity troponin I (hs-cTnI) over two years in comparison to a placebo (11).

Given the lack of SGLT2 expression in the myocardium, we hypothesized that SGLT2 inhibitors ameliorate HF by affecting specific mediators. A large-scale proteomic study has revealed considerable variations in plasma proteome in persons with HF undergoing SGLT2 inhibitors treatment (12). Whether SGLT2 inhibition regulates plasma proteins to improve HF symptoms remains unclear and is worth further exploration.

Mendelian Randomization (MR) is a genetic epidemiology strategy that uses genetic variations as instrumental variables (IVs) to assess the probable causal associations between exposure (risk factors) and clinical outcomes (13, 14). Because alleles linked with different traits are randomly assigned at conception, they are incredibly independent of confounders (15); thereby, the MR study significantly limits confounding. Additionally, the allocation of genetic variations precedes the onset of the outcome so that MR analysis could avoid potential reverse causal bias (16). Observational studies are often biased and confounded, which forces us to evaluate the causal association of SGLT2 inhibition and HF using the MR method. Moreover, the two-step MR is an extension of MR which is used to investigate whether mediating variables mediate the impacts of SGLT2 inhibition on HF.

In this study, we incorporated proteome-wide MR, numerous sensitivity tests, colocalization, and mediation evaluation to determine whether plasma proteins mediate the impact of SGLT2 inhibition on HF.

## 2 Methods

### 2.1 Study design

**Figure 1:**
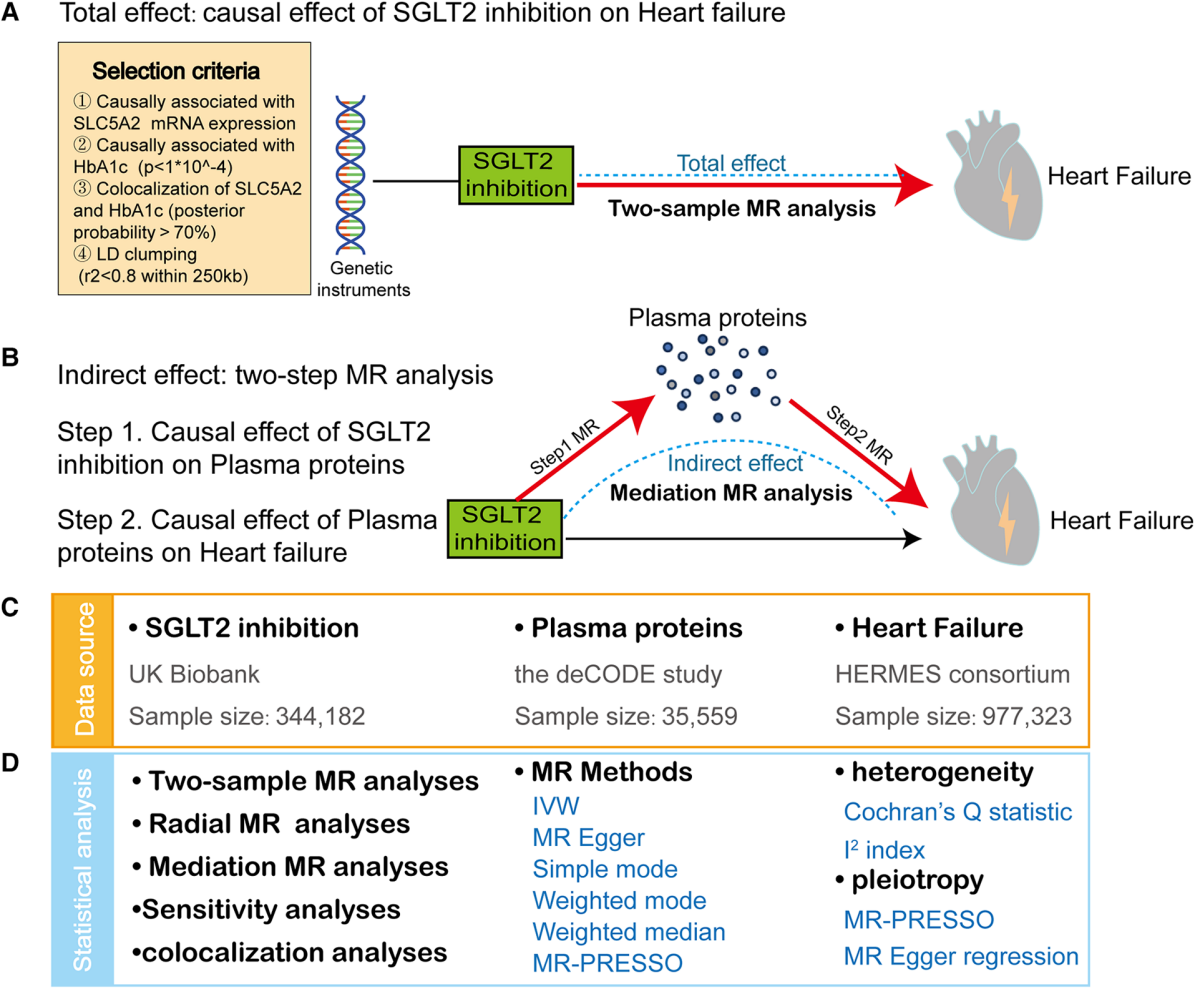
Study design and summary. (**A**) We applied a two-sample Mendelian Randomization (MR) analyses to reveal the causal effect of SGLT2 inhibition on heart failure (HF). The frame diagram also encompasses criteria for SGLT2 inhibition instrument selection. (**B**) We conducted a two-step MR approach to identify plasma proteins mediating the causal effect of SGLT2 inhibition on HF: First, we estimated the effect of SGLT2 inhibition on 4,907 plasma proteins using MR analyses (step 1 MR). Second, we estimated the effect of the SGLT2 inhibition-driven proteins on HF, again using MR analyses (step 2 MR). (**C**) The data sources of exposure (SGLT2 inhibition), mediator (plasma proteins) and outcome (heart failure). (**D**) The statistical analyses in this study include two-sample MR analyses, radial MR analyses, mediation MR analyses, sensitivity analyses and colocalization analyses. IVW, inverse-variance-weighted method. Sensitivity analyses include heterogeneity test and pleiotropy test.

Figure 1 depicts a framework design for this study. First, we performed a two-sample MR to investigate the link of causation between SGLT2 inhibition and HF. Then, a two-step MR was performed to assess whether possible plasma proteins mediate the effects of SGLT2 inhibition on HF. The MR analysis is based on three crucial assumptions (13): (1) the selected genetic variants are strongly linked to the exposure. (2) the selected genetic variants should not be associated with confounders. (3) the selected genetic variants affect outcomes only via exposure. The research was reported following the STROBE-MR guidelines (17). The fundamental characteristics of these summary-level genome-wide association studies (GWASs) data are outlined in Supplementary Table 1, which have recently become publicly accessible and ethical permission can be acquired in the prior studies (18, 19).

### 2.2 Genetic predictors for SGLT2 inhibition

The genetic variants mimicking SGLT2 inhibition were derived from Dai's and Xu's studies (20, 21). The four-step selection process of genetic predictors for SGLT2 inhibition was described in detail in the original study and simply exhibited in Figure 1. Six genetic variants with strong associations with SGLT2 inhibition were selected for further MR analysis (Supplementary Table 2).

### 2.3 Proteomic data source

The genetic associations with 4,907 proteins in the plasma proteome were derived from a large-scale pQTL(protein quantitative loci) research of 35,559 Icelanders (18). The SomaScan version 4 test (SomaLogic) was used to analyze the plasma samples. Age, sex, and sample age adjustments were made for each of the 4,907 examined aptamers using rank-inverse normal transformed levels. Following rank-inverse normal transformation, residuals were re-standardized, becoming the standardized phenotypes for genome-wide association testing conducted under the BOLT-LMM linear mixed model.

### 2.4 Outcome data source

The summary data of the genetic association with HF were obtained from the Heart Failure Molecular Epidemiology for Therapeutic Targets (HERMES) consortium (19). The HERMES study comprised 47,309 cases and 930,014 controls of European ancestry, including both 17 population cohort studies (38,780 HF cases, 893,657 controls) and 9 case-control studies (8,529 cases, 36,357 controls). The genetic information was imputed to the 1,000 Genomes Project (mainly), Haplotype Reference Consortium, or study-specific reference panels (19).

### 2.5 Genetic instrument selection

For MR analysis, the independent and significant IVs for exposure were extracted according to these criteria: *P *< 5e-8, r^2 ^< 0.001, the minimum allele frequency >0.01 and without linkage disequilibrium within 10 MB, based on the European 1,000 Genomes Project reference panel (22). Additionally, cis-PQTL (cis-acting protein quantitative loci) of leucine rich repeat transmembrane protein 2 (LRRTM2) were selected from single-nucleotide polymorphism (SNPs) within ± 500 Kb window around the transcription initiation site of the protein-coding gene at the genome-wide significance threshold (*P* < 5e-8) and without linkage disequilibrium within 10 MB.

### 2.6 MR analysis

The inverse-variance-weighted (IVW) approach was the major statistical model to assess the causal association of exposure on the outcome by weighted averaging the effect size of the instrumental variable estimates (23). For exposures with a single SNP, the Wald ratio approach was used. In addition, the weighted-median method is a commonly applied method for estimating causality in the presence of heterogeneity, as it permits departures from the Mendelian Randomization assumption for up to 50% of instrumental variable estimates. Simple mode and weighted mode methods were used for additional evaluations. We used scatter plots to depict the causal direction and forest plots to illustrate the effect size and statistical significance. We selected the false discovery rate (FDR) threshold for multiple tests at *P* = 0.05 as the significance level (24). The **“**TwoSampleMR” package was applied to perform MR evaluation.

### 2.7 Validation of MR assumptions and sensitivity analyses

The strength of the IVs was assessed using the F-statistic. $F=(n-2)\times \frac{R^{2}}{1-R^{2}}$, *n* = sample size, the coefficient of determination (R^2^) served as a metric to measure the proportion of variation explained by individual SNP, R^2^ is calculated using the following formula (25, 26): $R^{2}=\frac{2\times \beta^{2}\times \mathrm{EAF}\times (1-\mathrm{EAF})}{2\times \beta^{2}\times \mathrm{EAF}\times (1-\mathrm{EAF})+2\times SE^{2}\times n\times \mathrm{EAF}\times (1-\mathrm{EAF})}$, where EAF denotes the effect allele frequency, β is the estimated genetic effect size of the SNP, *n* = sample size, and SE signifies the standard error of the genetic effect size. F-statistic below 10 was considered weak IVs, which might cause biased results and would be excluded.

To avoid false positive results, we calculate the *P*-value of Cochran's Q statistic and I^2^ index to evaluate heterogeneity. *P* < 0.05 and I^2^ > 50% indicate substantial heterogeneity of IVs, a methodology applied in an earlier study (27). If heterogeneity existed, we applied a random-effects IVW model for MR analysis; if there was no apparent heterogeneity, we employed a fixed-effects IVW model. *P* < 0.05 of Cochran's Q test also indicates the existence of horizontal pleiotropy (occurs when IVs affect outcomes through pathways other than the exposure we are interested in), which could induce potential bias. However, vertical pleiotropy (IVs affect outcomes through pathways along the same causal chain) may not breach the assumptions of the MR study.

In addition, we tested the value of intercept term and intercept *P*-value from MR Egger regression and global test *P*-value of Mendelian Randomization Pleiotropy RESidual Sum Outlier (MR-PRESSO) to assess horizontal pleiotropy, with *P* < 0.05 suggesting that there exists theoretical pleiotropy of IVs. If the horizontal pleiotropy existed, we employed radial MR and MR-PRESSO to exclude outlying genetic variants. A “leave-one-out” analysis was used to evaluate the influence of an individual SNP on the overall causative estimate.

### 2.8 Mediation MR analysis

We conducted a two-step MR study to elucidate whether plasma proteins mediate the link between SGLT2 inhibition and HF. To determine the indirect mediation effect of SGLT2 inhibition on HF, we used the “product of coefficient” method (28, 29). We estimated the proportion of the total effect mediated by plasma proteins by dividing the indirect effect by the overall effect (β1 × β2/β3), where β1, β2, and β3 represent the effects of SGLT2 inhibition on the plasma proteins, plasma proteins on HF, and SGLT2 inhibition on HF, respectively. Standard errors were determined employing the delta method, and effect estimates were derived from a two-sample MR analysis.

### 2.9 Colocalization analysis

Colocalization analysis was used to determine if cis-pQTL for the LRRTM2 and HF shared the same causative variant in order to detect potential bias generated by confounding due to linkage disequilibrium. As previously outlined (30, 31), based on the Bayesian test, colocalization analyses provide posterior probabilities for five hypothesis testing: H_0_ (No associations with either trait), H_1_ (Associations with protein expression, not with HF), H_2_ (Associations with HF, not with protein expression), H_3_ (Associations with both traits, two independent SNPs), H_4_ (Associations with both traits, one shared SNP). When the posterior probability of H_4_ > 70% was considered significant evidence of colocalization, we utilized the ‘coloc’ R package to perform colocalization analyses with default arguments.

## 3 Results

### 3.1 The causal effects of SGLT2 inhibition on HF

**Figure 2:**
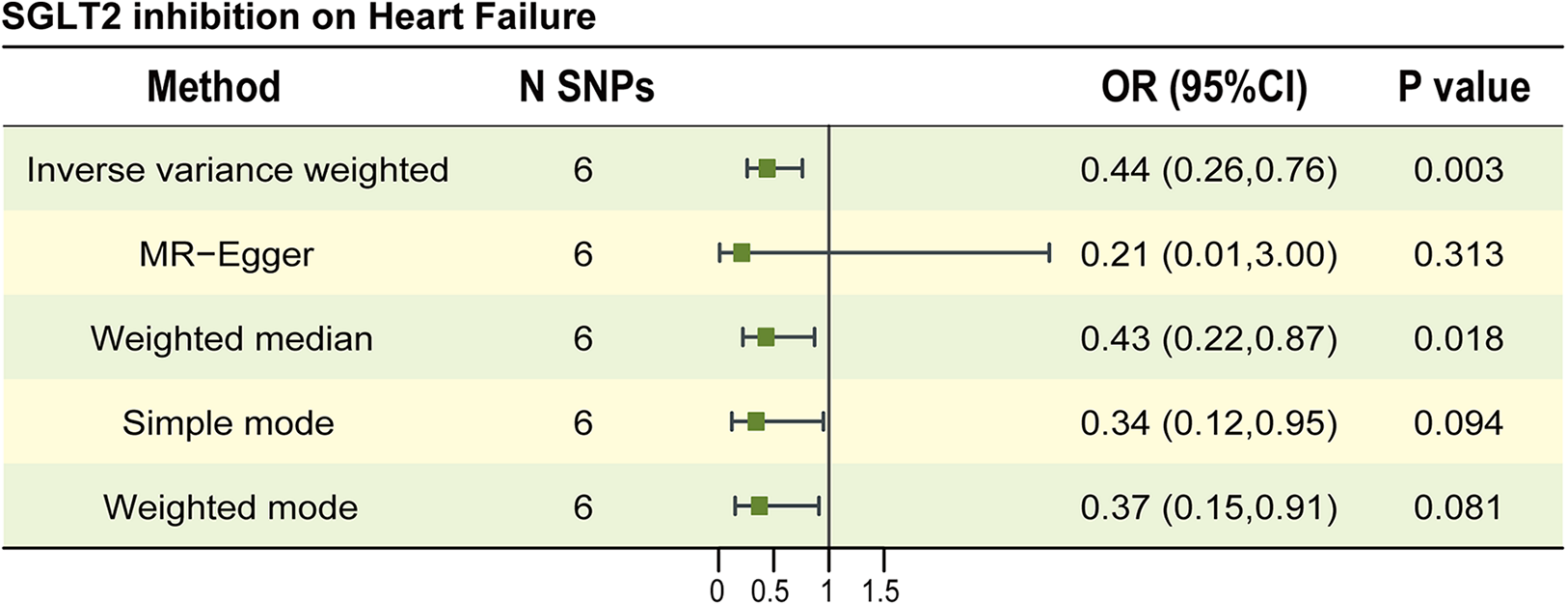
The causal estimates between SGLT2 inhibition on heart failure. The forest plot to visualize the causal effect of SGLT2 inhibition on Heart failure. IVW, inverse-variance-weighted method; OR, odds ratio; CI, confidence interval; SNP, single-nucleotide polymorphism.

The characteristics of SNPs representing SGLT2 inhibition were listed in Supplementary Table 2; the F statistics for all SNPs were larger than 10, indicating that all SNPs were strong IVs. MR study found a possible link between SGLT2 inhibition and a lower incidence of HF. The OR (95% CI, P) of the IVW method was 0.44 (0.26–0.76, *P* = 0.003) (Figure 2, Supplementary Table 3). Other statistical models displayed similar estimate directions for the SGLT2 inhibition on HF, suggesting the robustness of the main results (Figure 2, Supplementary Table 3). The scatter plot and forest plot of the correlation between SGLT2 inhibition and HF are illustrated in Supplementary Figures 1A,B, revealing consistent findings.

No significant horizontal pleiotropy was found in the MR Egger regression (Egger intercept = 0.009, *P* = 0.604) and MR-PRESSO (global test *P*-value = 0.901) method. In IVW and MR Egger analyses, the *P*-value of Q statistic was 0.880 and 0.834, respectively and the I^2^ < 50% in both methods, indicating no apparent sign of heterogeneity in estimating the impact of SGLT2 inhibition on HF. The data is shown in Supplementary Table 3. As suggested by leave-one-out analyses, the impact of SGLT2 inhibition on HF may not appear to be significantly swayed by any single SNP (Supplementary Figure 1C).

### 3.2 Two-step MR of SGLT2 inhibition, circulating proteins and HF

#### 3.2.1 SGLT2 inhibition to circulating proteins (step 1 MR)

**Figure 3:**
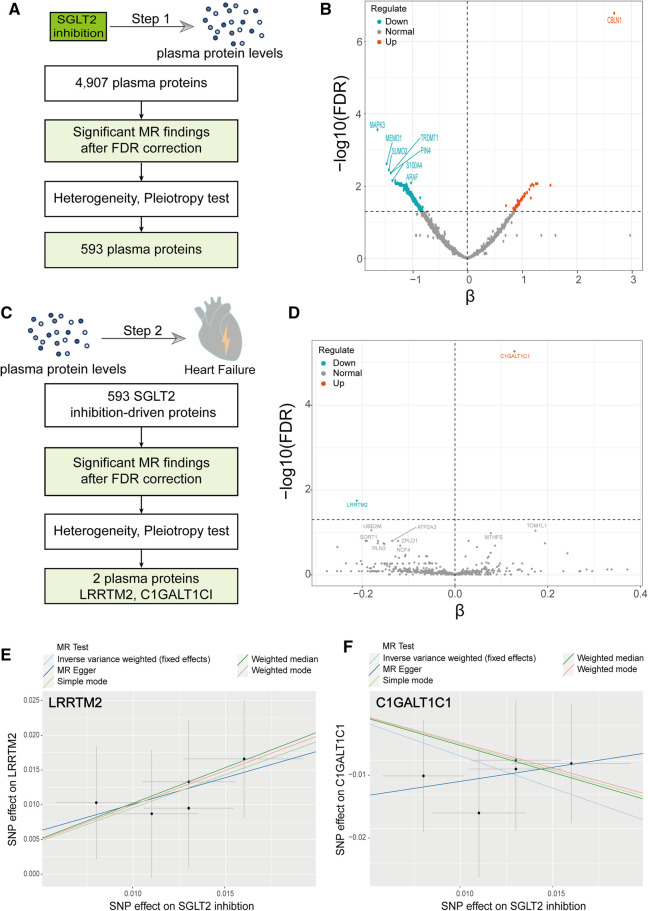
A Two-step MR analysis. (**A**) Flow diagram of step 1 MR analyses. (**B**) Volcano plot illustrating the effect of SGLT2 inhibition on each circulating protein from the MR analyses using the inverse-variance-weighted method. (**C**) Flow diagram of step 2 MR analyses. (**D**) Volcano plot illustrating the effect of SGLT2 inhibition-driven proteins on heart failure from the MR analyses using the inverse-variance-weighted method. (**E**) Scatter plot of the causal relationship between SGLT2 inhibition on LRRTM2. (**F**) Scatter plot of the causal relationship between SGLT2 inhibition on C1GALT1C1. The slope of each line represents the causal relationship of each method.

To assess the proteome-wide impact of SGLT2 inhibition, we conducted a two-sample MR with SGLT2 inhibition as the exposure and regarded 4,907 plasma proteins as the outcome. The analysis process was illustrated in Figure 3A. Among the 4,907 plasma proteins, we identified 593 proteins impacted by SGLT2 inhibition, mainly analyzed through the IVW method, using the FDR-corrected threshold of *P* < 0.05 (Figures 3A,B, Supplementary Table 4). In addition, we applied horizontal pleiotropy and heterogeneity tests to assess the stability of MR results. There was no apparent horizontal pleiotropy in FDR-significant proteins with all Egger intercept *P*-values greater than 0.05 in MR Egger regression and global test *P*-value greater than 0.05 in the MR-PRESSO test. In IVW and MR Egger analysis, the *P*-value of Q statistic was >0.05 and I^2 ^< 50% for all FDR-significant proteins, indicating no remarkable heterogeneity within the identified proteins (Supplementary Table 4).

#### 3.2.2 “SGLT2 inhibition”-driven proteins to HF (step 2 MR)

Subsequently, we explored the causal impacts of “SGLT2 inhibition”-driven proteins (obtained above) on outcomes of HF, employing a two-sample MR analysis again. We employed pQTLs of the 593 plasma circulating proteins as exposure and HF as the outcome. Among the 593 plasma proteins, we screened out two proteins that affect HF, mainly analyzed through the IVW method, utilizing the FDR-corrected threshold of *P* < 0.05, including LRRTM2 and C1GALT1C1 (Figures 3C-F, Supplementary Table 5).

#### 3.2.3 Analyses for SGLT2 inhibition to LRRTM2, C1GALT1C1 (step 1 MR)

**Figure 4:**
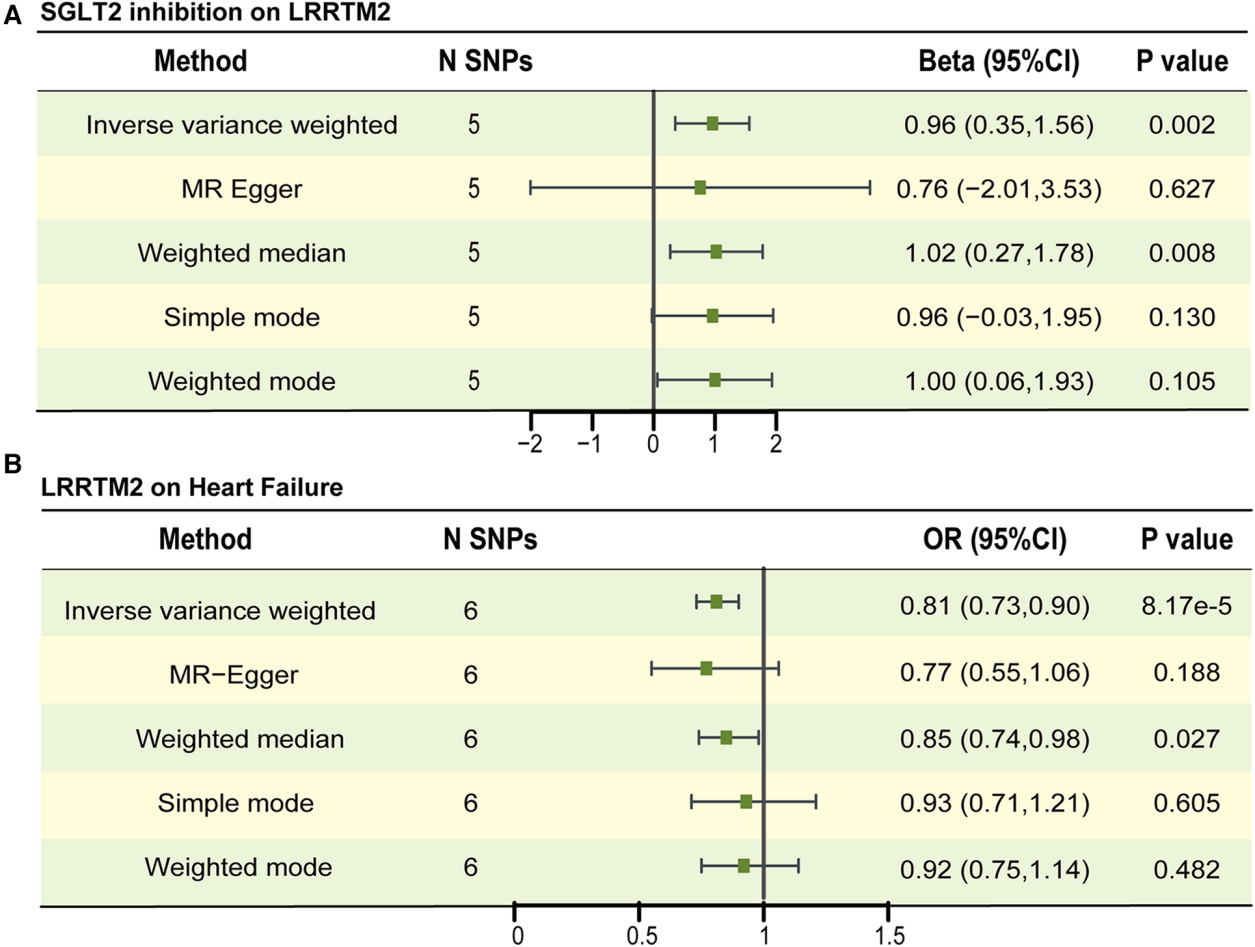
The causal estimates between SGLT2 inhibition on LRRTM2, LRRTM2 on heart failure. (**A**) The forest plot to visualize the causal effect of SGLT2 inhibition on LRRTM2. (**B**) The forest plot to visualize the causal effect of LRRTM2 on heart failure. IVW, inverse-variance-weighted method; OR, odds ratio; CI, confidence interval; SNP, single-nucleotide polymorphism.

We used a two-sample MR to explore the causal effect between SGLT2 inhibition on LRRTM2 and C1GALT1C1. MR analysis demonstrated that SGLT2 inhibition is positively related to LRRTM2 [β = 0.96, 95% CI (0.35, 1.56), *P* = 0.002] and negatively related to C1GALT1C1 [β = −0.94, 95% CI (−1.56, −0.32), *P* = 0.003] of the IVW method. We also use other methods including MR Egger, weighted-median, simple mode and weighted mode to ensure the robustness of the main results (IVW). Other statistical models displayed similar estimate directions for the SGLT2 inhibition on LRRTM2 but C1GALT1C1 (Figures 3E,F, 4A and Supplementary Table 6). For C1GALT1C1, although the IVW method showed a causal effect between SGLT2 inhibition and C1GALT1C1, the MR Egger method showed an opposite direction, suggesting that the causality was invalid. So, we chose LRRTM2 to proceed to step 2 MR analysis. The forest plot of the correlation between SGLT2 inhibition and LRRTM2 is illustrated in Supplementary Figure 2A.

No remarkable horizontal pleiotropy existed for MR analyses for SGLT2 inhibition to LRRTM2, Egger intercept = 0.003, *P* = 0.897 and global test *P*-value = 0.993 for LRRTM2. In IVW and MR Egger analyses, the *P* value of Q statistic was 0.990 and 0.964, respectively, and the I^2 ^< 50% in both methods, indicating no apparent sign of heterogeneity existed in estimating the influence of SGLT2 inhibition on plasma LRRTM2 level (Supplementary Table 6). Leave-one-out analyses suggest that the effect of SGLT2 inhibition on LRRTM2 was not noticeably impacted by any single SNP (Supplementary Figure 2B).

#### 3.2.4 Analyses for LRRTM2 to heart failure (step 2 MR)

In step 2 MR, pQTLs linked to LRRTM2 are utilized as the exposure, while heart failure (HF) is considered the outcome. We identified eight SNPs associated with LRRTM2 using a threshold of *P* < 5 × 10^−8^ and excluded SNPs with potential linkage disequilibrium. The selected SNPs are listed in Supplementary Table 7. The F-statistic surpassed 10 for all SNPs. MR study revealed that a one SD increase in genetically predicted LRRTM2 levels was related to decreased odds of HF [OR = 0.81, 95% CI (0.73, 0.90), *P* = 8.17 ×  10^−5^] for the IVW method. Other statistical models displayed similar estimate directions (Figure 4B, Supplementary Table 8, Supplementary Figure 3A). The scatter plot and forest plot of the correlation between LRRTM2 and HF are illustrated in Supplementary Figures 3A,B.

The sensitivity tests showed no apparent horizontal pleiotropy in the MR analyses involving LRRTM2 and HF. Egger intercept = 0.005, *P* = 0.751 and global test *P*-value = 0.261. In IVW and MR Egger analysis, the *P* value of Q statistic was 0.269 and 0.183 respectively, and the I^2^ < 50% in both methods, indicating there was no apparent heterogeneity in estimating the effect of plasma LRRTM2 level on HF (Supplementary Table 8). According to leave-one-out analyses, the influence of LRRTM2 on heart failure (HF) did not seem to be significantly affected by any individual SNP (Supplementary Figure 3C).

Next, we extracted cis-pQTL of LRRTM2 as exposure. Only one SNP was selected; the information is in Supplementary Table 9. The F-statistic of LRRTM2 cis-pQTLs IVs was 87.14. We employed a two-sample MR to analyze the association between the cis-pQTLs of LRRTM2 and HF. The Wald ratio approach was performed. The data showed that LRRTM2 levels were associated with a decreased odds ratio of HF [OR = 0.69, 95% CI (0.57, 0.84), *P* = 2.73  ×  10^−4^] (Supplementary Table 10).

### 3.3 Reverse MR analysis for heart failure to LRRTM2

A reverse-direction MR study was performed to evaluate the potential reverse causality of LRRTM2 and HF. Using a significance threshold of *P* < 5 × 10^−8^ and excluding those with potential linkage disequilibrium, we pinpointed 12 SNPs associated with HF (Supplementary Table 11). The F statistics for all SNPs surpassed 10, indicating that all SNPs were strong IVs. HF was related to LRRTM2 [β = −0.18, 95% CI (−0.31, −0.06), *P* = 2.8 × 10^−3^], which implies that HF may be negatively related to plasma LRRTM2 level. Other statistical methods displayed similar estimate directions. The scatter plot and forest plot of the correlation between HF and LRRTM2 reveal consistent findings (Supplementary Table 12, Supplementary Figures 4A,B).

The MR Egger regression (Egger intercept = −0.011, *P* = 0.413) and MR-PRESSO (global test *P*-value = 0.985) methods revealed no apparent sign of horizontal pleiotropy. In IVW and MR Egger analyses, the *P* value of Q statistic was 0.980 and 0.992 respectively, and the I^2^ < 50% in both methods, indicating there was no significant heterogeneity in estimating the effect of HF on plasma LRRTM2 level (Supplementary Table 12). As suggested by leave-one-out analysis, the impact of HF on LRRTM2 did not appear to be significantly swayed by any single SNP (Supplementary Figure 4C).

### 3.4 Mediation MR analysis linking SGLT2 inhibition With HF

Using a two-step MR with the “product of coefficient” method, we conducted a mediation analysis to analyze the mediation of plasma LLRTM2 levels between SGLT2 inhibition and HF. The fraction of mediation effects was computed by dividing the estimated LRRTM2-mediated impact by the estimated overall effect of SGLT2 inhibition on HF. The LRRTM2-mediated effect estimate was calculated by multiplying the impact of SGLT2 inhibition on the plasma proteome by the effect of circulating proteins on HF. The data shows that plasma LRRTM2 levels partially mediated the overall effect of SGLT2 inhibition on HF [proportion mediated = 24.63%, 95% CI (7.32%, 47.56%), *P* = 0.02] (Table 1).

**Table 1:** The mediation effect of SGLT2 inhibition on HF via LRRTM2.

Mediator	Total effect β (95% CI)	Direct effect A β (95% CI)	Direct effect B β (95% CI)	Mediation effect β (95% CI)	*p*	Mediated proportion (%) (95% CI)
LRRTM2	−0.82 (−1.36, −0.28)	0.96 (0.35, 1.56)	−0.21 (−0.32, −0.11)	−0.20 (−0.39, −0.06)	0.02	24.63 (7.32, 47.56)

“Total effect” indicates the effect of SGLT2 inhibition on HF, “direct effect A” indicates the effect of SGLT2 inhibition on LRRTM2, ‘direct effect B’ indicates the effect of LRRTM2 on HF and “mediation effect” indicates the effect of SGLT2 inhibition on HF through LRRTM2. Total effect, direct effect A and direct effect B were derived by IVW; mediation effect was derived by using the delta method. All statistical tests were two-sided. *P* < 0.05 was considered significant.

### 3.5 Colocalization analysis

**Figure 5:**
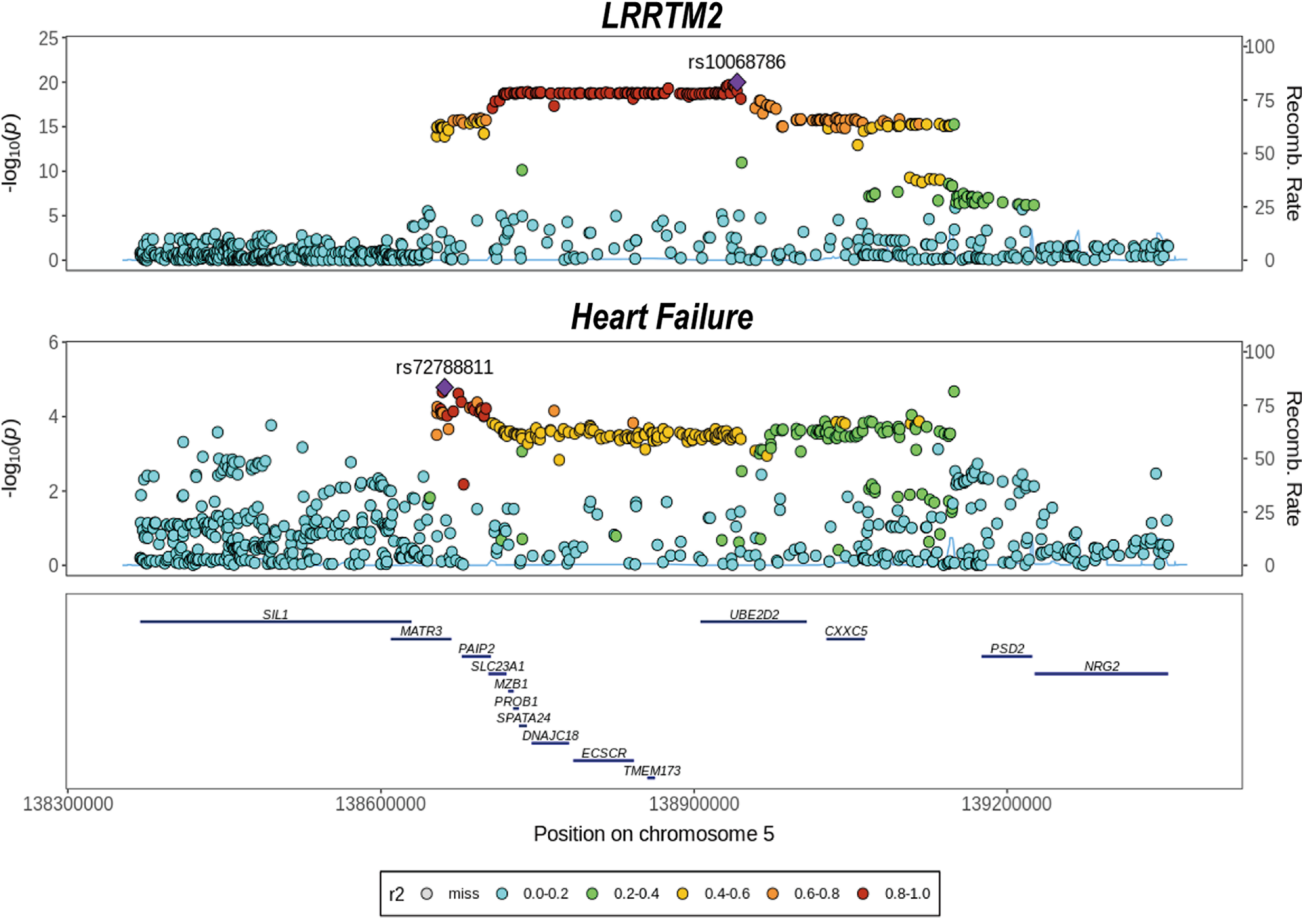
Colocalization analysis of cis-pQTL for LRRTM2 with heart failure. We evaluated whether the cis-pQTL for LRRTM2 shared the same causal variant with heart failure outcome using colocalization.

Considering that the two-step MR analyses implicated LRRTM2 as a potential mediator involving the impact of SGLT2 inhibition on HF. We used colocalization analyses to determine whether the cis-pQTLs for LRRTM2 shared had the same unique causative mutation with HF. This can be used to determine whether LD influenced MR analyses. The results showed that LRRTM2 had a high posterior probability of colocalization with HF (PP.H4 = 0.72, Figure 5). These findings back up the results of Mendelian Randomization and reveal that LRRTM2 and HF share the same causal variant (Supplementary Table 13).

## 4 Discussion

In this study, MR analysis unveiled a negative causality between SGLT2 inhibition and HF. Employing a two-step MR, we identified LRRTM2 among 4,907 plasma proteins, implying that SGLT2 inhibition may influence HF through its impact on circulating LRRTM2 levels. Our data showed that SGLT2 inhibition positively correlated with circulating LRRTM2 levels, and the LRRTM2 levels were negatively correlated with HF. Furthermore, the colocalization analysis for cis-pQTLs of LRRTM2 and HF indicated a shared causal variant, reinforcing the connection between LRRTM2 and HF.

LRRTM2 (leucine-rich repeat transmembrane neuronal 2), a member of the LRRTMs (Leucine-rich repeat transmembrane neuronal proteins) family, is a synaptogenic receptor protein, located mainly in the postsynaptic membrane (32, 33). The interaction of LRRTM2 and presynaptic receptors Neurexin-1 (NRXN1) plays a critical role in regulating excitatory synapse development (34, 35). A study defined a subset of neuronal cells expressing neural cell adhesion proteins NRXN1, NRXN3 and NCAM2 that were located in all four compartments of the heart (36). Importantly, Umair Sajid et al. found that the mutations of the neurexin-1 (NRXN1) gene caused idiopathic dilated cardiomyopathy (37). These findings reveal that LRRTM2 may affect heart disease by regulating NRXN1.

In the heart, cardiac ganglionated plexi (GPs) reside in fat pads, constituting the “intrinsic cardiac nervous system” (38). The neural regulation of the heart involves the coordinated interplay of the sympathetic and vagus nervous systems (39, 40). These systems, through neurotransmitter release, influence the contraction and relaxation of the heart to maintain its normal function. Heart failure is a syndrome characterized by impaired heart pumping function, resulting in an inadequate cardiac output to meet the basic metabolic needs of body tissues due to diverse factors. The critical pathogenesis of HF has been recognized for decades, involving autonomic imbalance and hormonal hyperactivity. The observed autonomic dysfunction is considered a common manifestation of HF, arising from alterations in cardiac function-related hemodynamics. The concept of autonomic imbalance was suggested in the 1990s as a direct contributor to HF progress (41). In the study of Tucker, et al., they defined a subset of neuronal cells expressing neural cell adhesion proteins NRXN1 in the heart and speculated this subcluster cells may originated from the intrinsic cardiac autonomic neural network (42). Therefore, we hypothesized that LRRTM2 regulates the intrinsic cardiac autonomic system in the heart by interacting with NRXN1, thereby improving HF.

Several studies have reported that SGLT2 inhibitors could improve the function of pancreatic β cells (43, 44). In the study of Fu et al., they found I-BET151 (a small-molecule inhibitor of a family of bromodomain-containing transcriptional regulators) could also protect pancreatic β cells, which may be related to elevated LRRTM2 (45). These findings suggest that SGLT2 inhibitors may have a regulatory effect on LRRTM2. In addition to acting on SGLT2 protein, SGLT2 inhibitors also regulate other proteins. Yang et al. reported that SGLT2 inhibitor Dapagliflozin attenuates cardiac fibrosis and inflammation by reverting the HIF-2α signaling pathway in arrhythmogenic cardiomyopathy (46). Lin et al. found that Dapagliflozin (an SGLT2 inhibitor) against obesity related cardiomyopathy via NHE1/MAPK signaling (47). In our study, the MR results also revealed the causal effect of SGLT2 inhibition on upregulated plasma LRRTM2 levels. In summary, LRRTM2 may be a new target of SGLT2 inhibitors.

The bidirectional causal association between SGLT2 inhibition and HF sparked our contemplation. On the one hand, elevated LRRTM2 could promote the formation of excitatory synapses, thereby improving autonomic imbalance in the heart and ultimately reducing the risk of HF. On the other hand, the insufficiency of oxygen caused by heart failure can directly impact energy metabolism and gene expression in nerve cells, which may decrease the level of LRRTM2. However, more study is needed to investigate the particular mechanism of a bidirectional causal link SGLT2 inhibition and HF.

Several limitations require acknowledgment. First, the MR and colocalization studies were limited to people of European origin to reduce potential biases caused by population stratification and different genetic connections among ancestries. However, whether LRRTM2 mediates the impact of SGLT2 inhibition on heart failure in non-European populations warrants further exploration. Second, Due to the unavailability of datasets for gender stratification and disease classification, we refrained from conducting Mendelian Randomization analyses on both gender stratification and heart failure classification. Finally, despite conducting diverse sensitivity analyses and applying rigorous criteria, as is common in all MR studies (48, 49), dealing with horizontal pleiotropy in the MR setting was challenging.

Despite these limitations, our findings provide some direction for future research. Firstly, this study expands on the mechanism of SGLT2 inhibitors in improving heart failure and provides a new idea for exploring the basic research on SGLT2 inhibitors in improving heart failure. Secondly, because plasma proteins are easy to measure, the levels of plasma LRRTM2 could serve as an indicator of the efficacy of SGLT2 inhibitors in treating HF. Its level could also indicate the severity of heart failure. Lastly, given that LRRTM2 is a protein involved in synaptic formation, it suggests that SGLT2 inhibitors may have a certain regulatory effect on neurological diseases, thereby broadening the clinical application of SGLT2 inhibitors. Overall, the findings of this study offer new insights for future clinical and basic research.

## 5 Conlusion

In summary, we employed a two-step MR, sensitivity tests, colocalization, and mediation analyses, revealing LRRTM2 as a significant mediator in the impact of SGLT2 inhibition on HF. This study provides fresh insights into the influence of SGLT2 inhibition on HF and introduces a new perspective for HF treatment exploration.

## Data Availability

The original contributions presented in the study are included in the article/**Supplementary Material**, further inquiries can be directed to the corresponding authors.
