# Supplementary material for: SGLT2 inhibition, plasma proteins, and heart failure: a proteome-wide Mendelian Randomization and colocalization study
Source: Front Cardiovasc Med. 2024 Apr 25;11:1371513. doi: 10.3389/fcvm.2024.1371513 (PMC11079590; doi:10.3389/fcvm.2024.1371513)
Supplement: Supplementary file 1 [file Datasheet1.docx]

Supplementary Material

## Supplementary Figures


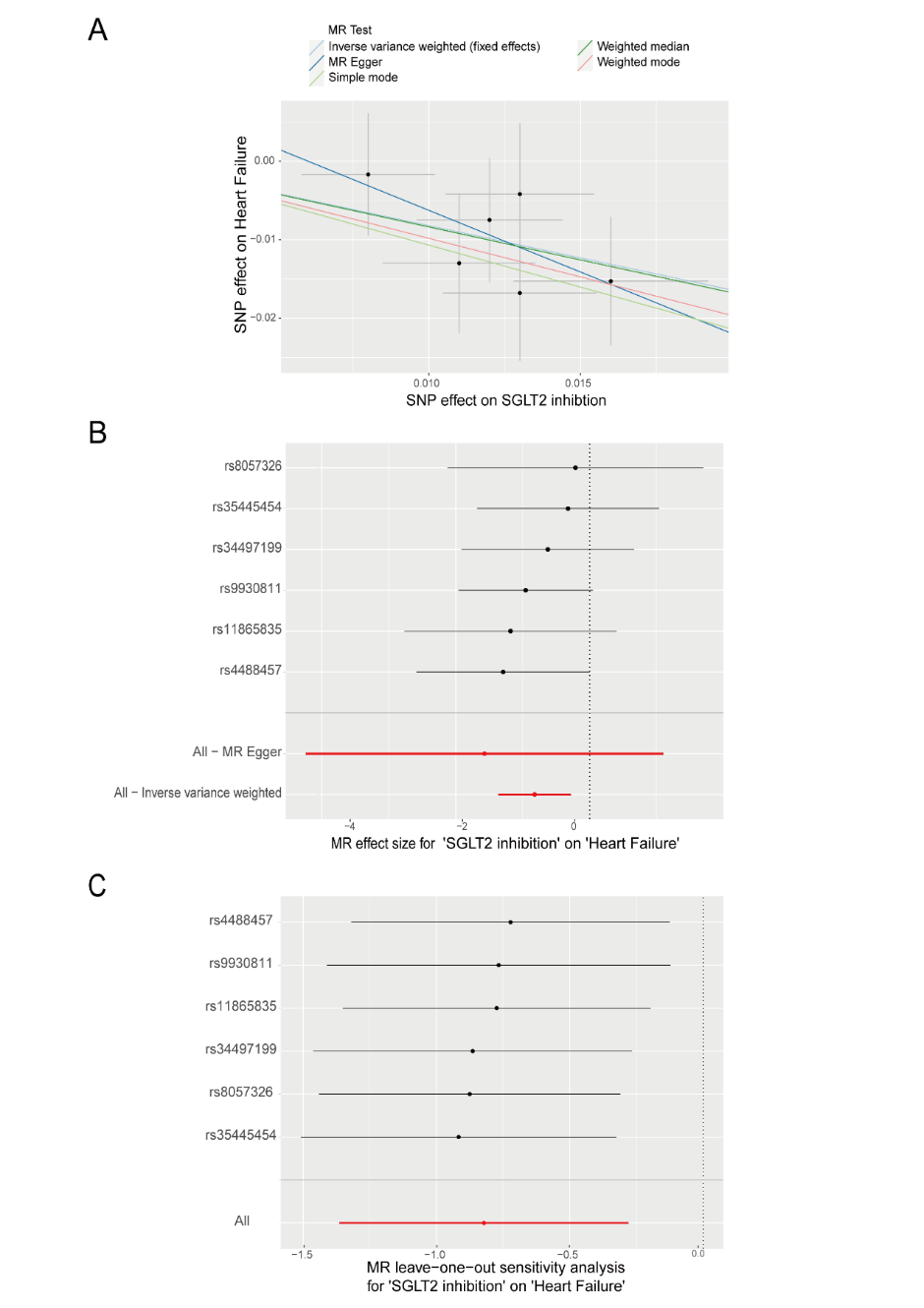


**Supplementary Figure 1.** **MR analysis for the effect of SGLT2 inhibition on heart failure.**

**A.** Scatter plot of the causal relationship between SGLT2 inhibition and heart failure. The slope of each line represents the causal relationship of each method. **B.** Forest plot to visualize the causal effect of every single SNP of SGLT2 inhibition on Heart failure. **C.** Leave-one-out plot of the causal relationship between SGLT2 inhibition and Heart failure.


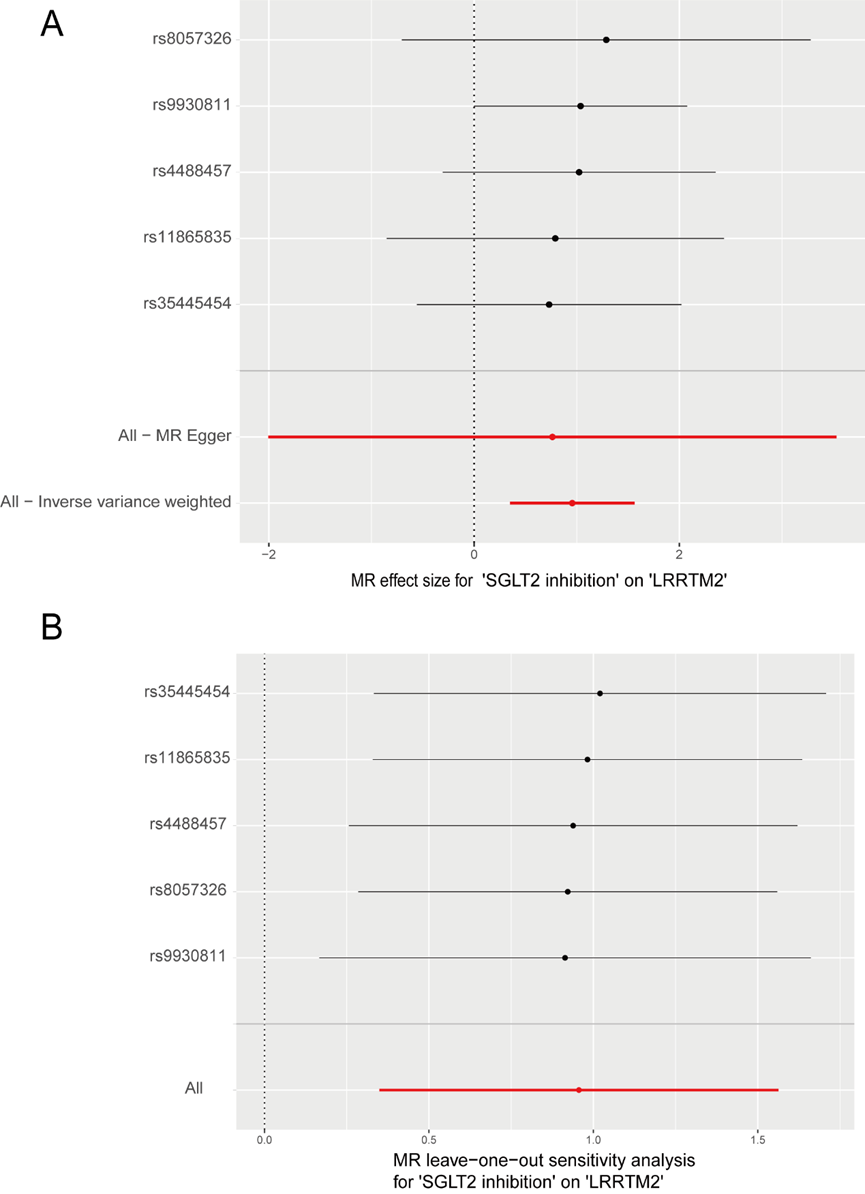


**Supplementary Figure 2.** **MR analysis for the effect of SGLT2 inhibition on LRRTM2.**

**A.** Forest plot to visualize the causal effect of every single SNP of SGLT2 inhibition on LRRTM2. **B.** Leave-one-out plot of the causal relationship between SGLT2 inhibition and LRRTM2.


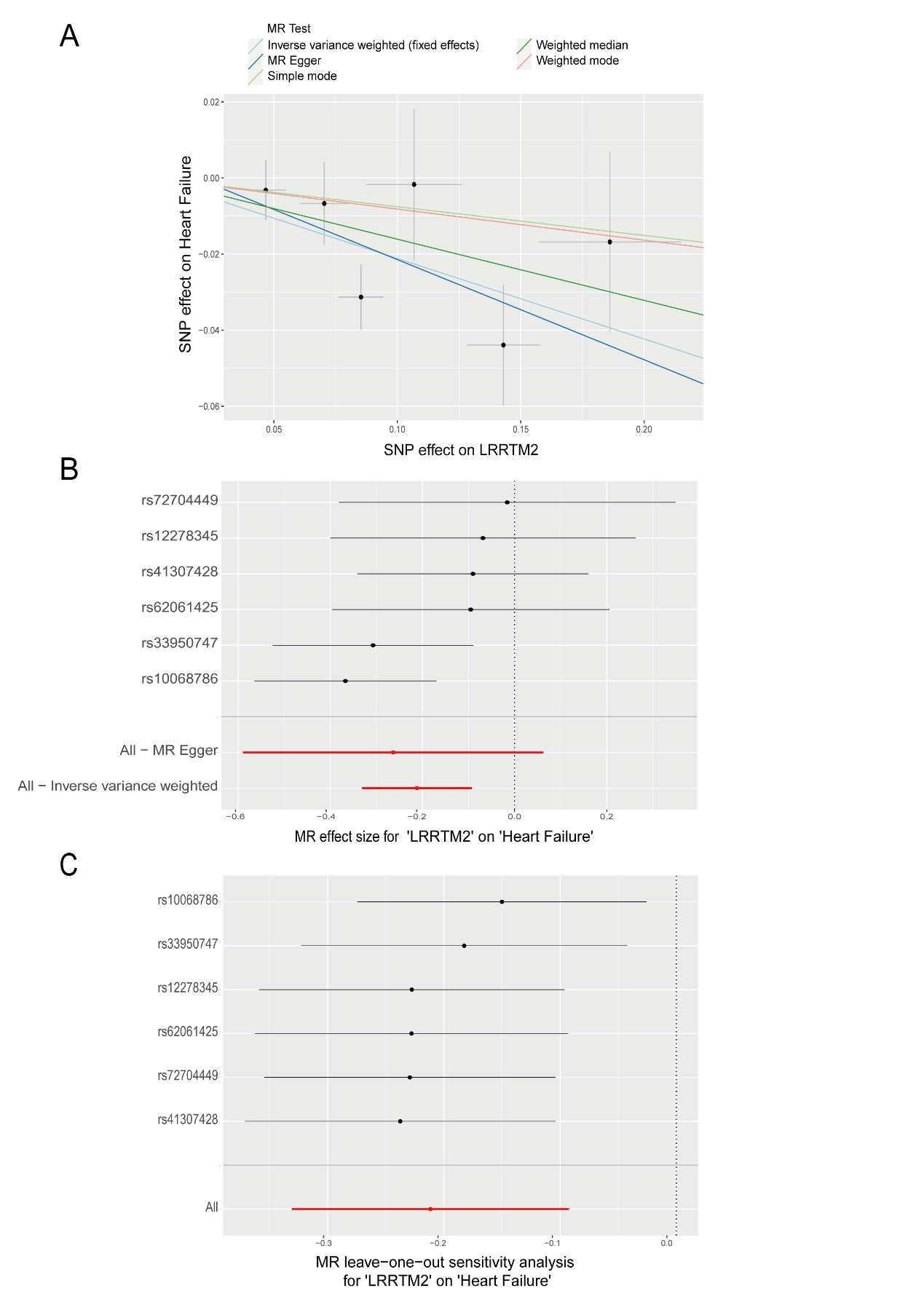


**Supplementary Figure 3.** **MR analysis for the effect of LRRTM2 on heart failure.**

**A.** Scatter plot of the causal relationship between LRRTM2 and heart failure. The slope of each line represents the causal relationship of each method. **B.** Forest plot to visualize the causal effect of every single SNP of LRRTM2 on heart failure. **C.** Leave-one-out plot of the causal relationship between LRRTM2 and heart failure.


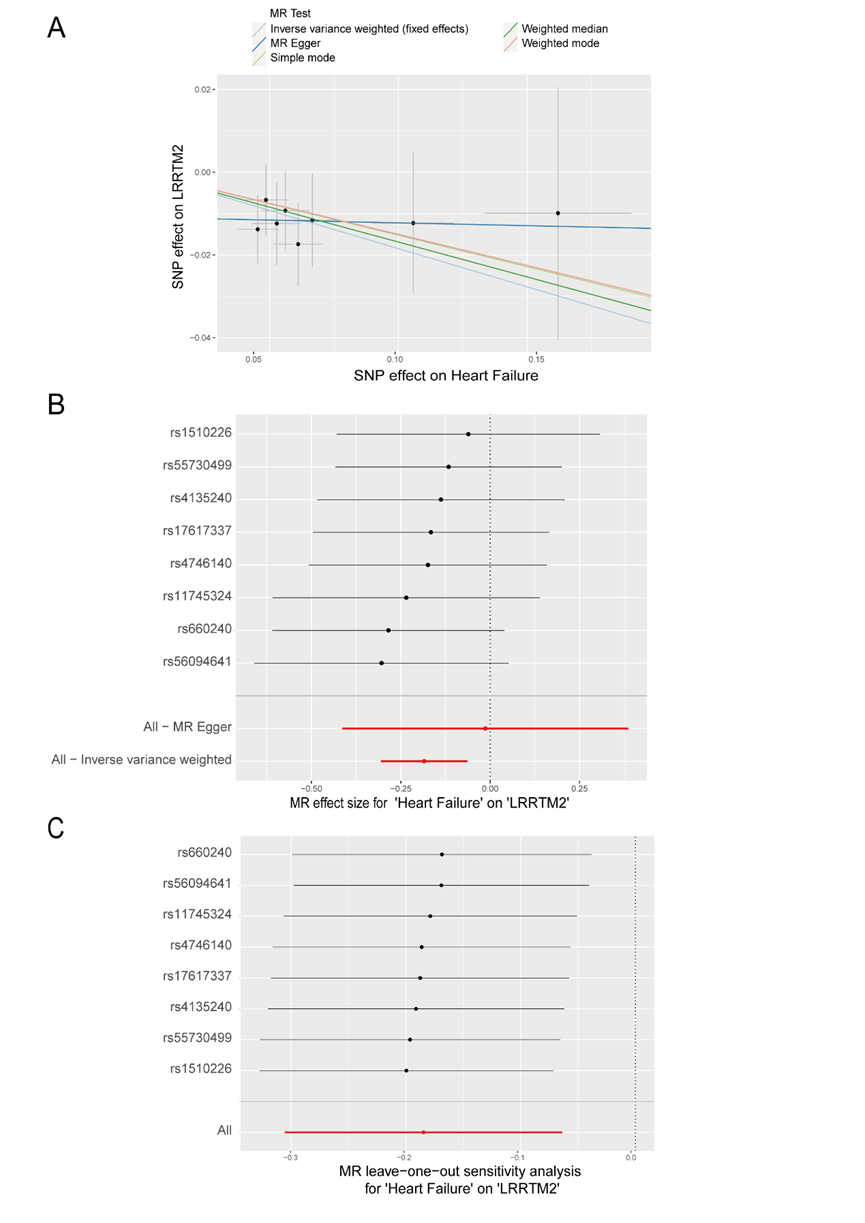


**Supplementary Figure 4.** **MR analysis for the effect of heart failure on LRRTM2.**

**A.** Scatter plot of the causal relationship between Heart failure and LRRTM2. The slope of each line represents the causal relationship of each method. **B.** Forest plot to visualize the causal effect of every single SNP of heart failure on LRRTM2. **C.** Leave-one-out plot of the causal relationship between heart failure and LRRTM2.
